# The Genetic Landscape of Familial Hypercholesterolemia in Telangana, Southern India: Novel Mutations and Clinical Implications

**DOI:** 10.7759/cureus.99069

**Published:** 2025-12-12

**Authors:** Supriya Garapati, Sakthivadivel Varatharajan, Ariyanachi K, Kishore Yadav, Rohit Saluja, Sailu Yellaboina

**Affiliations:** 1 Anatomy, All India Institute of Medical Sciences, Bibinagar, Hyderabad, IND; 2 Internal Medicine, All India Institute of Medical Sciences, Bibinagar, Hyderabad, IND; 3 Community and Family Medicine, All India Institute of Medical Sciences, Bibinagar, Hyderabad, IND; 4 Biochemistry, All India Institute of Medical Sciences, Bibinagar, Hyderabad, IND

**Keywords:** dutch lipid clinic network criteria, exome sequencing, familial hypercholesterolemia, next-generation sequencing, single nucleotide polymorphisms

## Abstract

Background: Familial hypercholesterolemia (FH) is a genetic disorder characterized by elevated low-density lipoprotein cholesterol (LDL-C) levels, leading to premature cardiovascular disease (CVD). This study aimed to identify genetic variants associated with FH in patients from Telangana State, India.

Methods: Probands with suspected FH were identified using the Dutch Lipid Clinic Network (DLCN) score, followed by cascade screening of their first-degree relatives. Targeted exome sequencing and pedigree analysis were performed to identify FH-associated genetic variants.

Results: We identified both novel and known high-impact mutations in genes implicated in FH pathogenesis, including stop-gain mutations in LPL (6/30; 20%) and LDLR (4/30; 13.3%), as well as splice donor site mutations in SLCO1B1 (1/30; 3.3%) and CETP (3/30; 10%). Notably, a novel frameshift mutation in LDLR was identified in two siblings (2/30; 6.7%), one of whom (50%) exhibited a homozygous variant and met the "Definite FH" classification based on the DLCN criteria. Additionally, moderate-impact variants rs2075291 (APOA5) and rs193922571 (LDLR) showed strong correlations with the DLCN score, suggesting increased susceptibility to FH. In contrast, rs6756629 (ABCG5) and rs11887534 (ABCG8) were strongly negatively correlated with LDL-C levels and the DLCN score, indicating potential protective effects against FH.

Conclusions: These findings highlight the genetic heterogeneity of FH and emphasize the importance of identifying novel pathogenic variants. Moreover, the study underscores the role of moderate-impact variants in FH susceptibility. Overall, this research enhances our understanding of the genetic landscape of FH in the Indian population, with implications for improved diagnosis, risk assessment, and personalized management.

## Introduction

Familial hypercholesterolemia (FH) is primarily an autosomal dominant disorder of cholesterol metabolism characterized by elevated low-density lipoprotein cholesterol (LDL-C) levels from an early age, predisposing individuals to premature atherosclerotic cardiovascular disease [1]. Early diagnosis and intervention are critical for mitigating FH-associated comorbidities and reducing cardiovascular mortality. The identification of an individual with FH often leads to the diagnosis of other first-degree relatives at risk, creating a positive cascade effect. Despite the widespread availability of cholesterol testing, FH remains largely underdiagnosed in the general Indian population, with many patients being diagnosed only at the time of hospitalization [2]. It is estimated that more than 95% of individuals with FH worldwide remain undiagnosed and untreated.

FH is a common genetic disorder that affects approximately one in 311 individuals globally. Its prevalence is similar across different regions and is notably higher in people with atherosclerotic cardiovascular disease (ASCVD) [3]. India bears a substantial burden of cardiovascular disease (CVD)-related deaths, accounting for one-fifth of the global total, particularly among the younger population [4]. A global burden of disease study revealed an alarming age-standardized CVD death rate of 272 per 100,000 people in India, surpassing the global average of 235 [5].

Most FH cases are attributed to defects in the LDL receptor (LDLR) or apolipoprotein B-100 (APOB) genes, whereas gain-of-function variants in the PCSK9 gene leading to its overproduction are less common [6]. Additional variants in genes such as APOE have been sporadically linked to the FH phenotype [7]. Homozygous FH can also arise from variants in the LDLRAP1 gene associated with an autosomal recessive form of the disease [8]. The severity of FH is influenced by the type of LDLR gene variant, with "null variants" resulting in severely reduced LDL receptor activity and a more severe phenotype, whereas "defective variants" retain some receptor function [9]. Pathogenic variants in the LDLR gene are the most prevalent cause of FH, exhibiting a wide spectrum of variations across populations.

In addition to genetics, nongenetic factors such as age, sex, and lifestyle also influence LDL-C levels. Studies have shown that females with FH tend to have higher levels of total cholesterol, LDL-C, and high-density lipoprotein cholesterol (HDL-C) than males do, although both sexes present lower HDL-C levels than individuals without FH do [10]. The average age of onset of coronary symptoms is delayed in females (55 years) compared with males (48 years) [11]. In men, lower HDL-C levels and a history of smoking are associated with an increased risk of developing coronary artery disease (CAD).

The prevalence of FH is estimated to be one in 250 individuals worldwide, but its identification and management remain suboptimal in many regions, particularly in developing countries. Early diagnosis and treatment are crucial for reducing the risk of cardiovascular complications associated with FH, such as premature coronary artery disease, myocardial infarction, and stroke. Recent advancements include the development of a 12-SNP LDL-C "SNP score" on the basis of common variants associated with elevated LDL-C levels [12]. This score has been validated in various populations and shows promise in identifying individuals with FH who lack identifiable mutations.

In India, limited data are available on the genetic epidemiology and mutational spectrum of FH. In this work, we characterize the genetic variants associated with FH in people from Telangana state, India. By combining targeted exome sequencing and detailed pedigree analysis, we identified novel and known high-impact mutations in genes implicated in FH pathogenesis. The identification of disease-causing mutations and the elucidation of familial inheritance patterns are crucial steps toward improving the diagnosis, risk stratification, and personalized management of FH in the Indian population. Furthermore, this study contributes to the growing body of knowledge on the genetic architecture of FH across diverse ethnic groups, facilitating a deeper understanding of the etiology of FH and potential therapeutic targets. The development of prognostic tools to detect FH in the population, especially in newborns and children, represents an unprecedented opportunity to initiate early treatment and reduce the burden of cardiovascular disease associated with FH.

The primary objective of this study was to identify and characterize genetic variants associated with familial hypercholesterolemia in patients from Telangana State, India, using targeted exome sequencing. Secondary objectives included: (1) determining the spectrum of high-impact and moderate-impact variants in known FH-associated genes; (2) analyzing familial inheritance patterns through comprehensive pedigree analysis; and (3) correlating identified genetic variants with clinical phenotypes as assessed by Dutch Lipid Clinic Network Criteria (DLCNC). This work aims to contribute foundational data on the genetic architecture of FH in the Indian population, with potential implications for future diagnostic and therapeutic strategies. This article was previously published as a preprint [13].

## Materials and methods

Study design and participant recruitment

This exploratory, cross-sectional study employed a family-based recruitment strategy with cascade screening of first-degree relatives to identify and characterize FH cases at AIIMS Bibinagar and healthcare centers in the Yadadri Bhuvanagiri district, Telangana, India. The final sample of 30 individuals from 15 families was determined by: (1) availability of probands meeting DLCNC within our regional healthcare catchment area during the study period, (2) willingness of first-degree relatives to participate in cascade screening, and (3) feasibility constraints including resources for targeted exome sequencing. This sample size is consistent with similar regional genetic characterization studies of rare Mendelian disorders and prioritizes depth of phenotypic and pedigree characterization over large sample size, enabling detailed analysis of variant segregation patterns within families.

Eligibility criteria included asymptomatic children and adolescents (0-20 years old) with LDL cholesterol exceeding 160 mg/dL or having a first-degree relative with elevated cholesterol and premature coronary heart disease (men <50 years, women <60 years). Clinical features such as tendon xanthomas at any age, arcus corneae younger than 55 years, or xanthelasma younger than 25 years were also considered. Individuals with a history of diabetes mellitus, hypothyroidism, obesity, alcohol use, smoking, or metabolic syndrome were excluded. Lipid profiling was performed using an XL 1000 analyzer with daily calibration using manufacturer-provided standards and quality control materials.

Proband identification and familial screening

Patients were prioritized on the basis of the DLCNC [14] for familial hypercholesterolemia, without considering genetic mutations [15]. A positive diagnosis according to any one of the criteria established a patient as a probable proband. Upon proband identification, informed consent was obtained for the screening of their first-degree relatives (parents, siblings, and children).

Cascade screening

A cascade screening approach was implemented to systematically identify individuals at risk for FH within the families of identified probands. This strategy involved the sequential screening of first-degree relatives (parents, siblings, and children) of each proband diagnosed with FH. Upon identification of a new FH case among these relatives, the cascade screening process was reiterated, with the newly diagnosed individual becoming a proband for subsequent screening of their first-degree relatives. This iterative process aims to maximize the detection of at-risk individuals within families affected by FH.

Sample collection, library preparation, and sequencing

Peripheral blood samples from patients with familial hypercholesterolemia were collected in EDTA tubes and stored at -80°C. DNA was extracted, quantified, and assessed for purity and integrity. DNA samples were required to meet the following quality control criteria: concentration ≥20 ng/μL (measured by Qubit fluorometry), A260/A280 ratio between 1.8-2.0, and DNA Integrity Number (DIN) ≥7.0 as assessed by Agilent TapeStation (Agilent Technologies, Inc., Santa Clara, CA, USA). Thirty samples that passed quality control were processed to create whole-genome libraries via the Twist Library Preparation EF Kit (Twist Bioscience, South San Francisco, CA, USA), followed by exome capture. The enriched libraries were subjected to target enrichment via the Twist Comprehensive Exome Panel. The final libraries were quality checked, quantified, pooled, diluted, and sequenced on the Illumina NovaSeq 6000 system (Illumina, Inc., San Diego, CA, USA), generating 150 bp paired-end reads. Raw read quality was rigorously assessed; all samples achieved a mean Phred quality score >35 (Range: 35.9-36.4). The percentage of bases with a Quality Score >30 (%Q>30) was consistently high, averaging >95% across all samples (Range: 94.6%-97.5%), ensuring high-confidence base calling for downstream variant analysis.

Exome sequence analysis and variant annotation

Fastp software v0.23.2 [16] with default parameters was used to process paired-end raw FASTQ files as part of a quality control and cleaning pipeline in next-generation sequencing (NGS) analysis. This included the automatic detection and trimming of adapter sequences often introduced during library preparation. To ensure high-quality data for downstream analysis, fastp filtered out reads falling below a specified quality threshold or containing a high percentage of low-quality bases. Paired-end reads in FASTQ format, previously cleaned and quality controlled via fastp, were aligned to the human reference genome, GRCh38.p14 (https://ftp.ncbi.nlm.nih.gov/genomes/), via the Burrows-Wheeler Aligner (BWA-MEM v0.7.17 with default parameters) algorithm [17].

The Picard tool v2.27.4 (AddOrReplaceReadGroups) was used to modify the Sequence Alignment Map (SAM) file, adding read group information essential for downstream analysis tools [18]. These tags provide crucial details about the sequencing data's origin and processing, aiding in quality control, alignment analysis, and variant calling. The command samtools sort [19] sorts the Binary Alignment Map (BAM) file by genomic coordinates, a critical step for many downstream analyses that require sorted input data for efficient processing, such as variant calling and visualization. The Picard tool MarkDuplicates was then employed to identify and mark potential PCR duplicates within the sorted BAM file. PCR duplicates, which arise during library preparation, can skew downstream analyses. The tool flags duplicate reads in the output BAM file and generates a metric file with statistics at the level of duplication.

The human reference genome (GRCh38.p14) and a known variant file (variant call format (VCF)) were indexed via ‘samtools faidx’ and the Genome Analysis Toolkit's (GATK) (v4.3.0.0) IndexFeatureFile [20] to optimize data access and processing efficiency during variant calling. GATK's BaseRecalibrator tool generated a base quality score recalibration (BQSR) table for the aligned sequencing data. This crucial step corrects systematic errors in base quality scores that can arise from sequencing technologies and library preparation, improving downstream variant calling accuracy. The recalibration table, which is specific to the input dataset, was created by referencing the human genome (GRCh38.p14) and incorporating information from a known variant file (VCF). This process ensures that base quality scores more accurately reflect the true probability of sequencing errors, enhancing the reliability of subsequent analyses.

The GATK tool ApplyBQSR then recalibrated the base quality scores in the aligned and duplicate-marked BAM files. By utilizing the BQSR table and the human reference genome (GRCh38.p14), the base quality scores in the BAM file were adjusted to reflect the true probability of sequencing errors more accurately, improving the accuracy and reliability of downstream variant calling and analysis. The GATK tool HaplotypeCaller performs variant calling on the recalibrated BAM file, utilizing the human reference genome (GRCh38.p14). This process involved local reassembly of haplotypes and identification of potential variants in the dataset. The GATK tool GenotypeGVCFs then performs joint genotyping on the genomic VCF (gVCF) file produced by HaplotypeCaller. The human reference genome (GRCh38.p14) was used to consolidate genotype likelihoods across all genomic positions, resulting in a final VCF file with high-confidence variant calls. Compared with analyzing individual samples independently, this approach enhances variant detection accuracy and sensitivity. The GATK tool VariantFiltration was then applied to filter out potentially low-quality variant calls from the VCF file. Using the human reference genome (GRCh38.p14), variants were assessed on the basis of specific quality metrics, and filters were applied to remove variants not meeting quality thresholds.

The BCFtools reheader command updated the header information in each original VCF file to reflect the sample source. Then, the BCFtools index command is used to index each VCF file for efficient random access to specific variants. Finally, the BCFtools merge [21] command was used to combine the final VCF files of all samples into a single merged and compressed VCF file. This merged file was then indexed via GATK's IndexFeatureFile for efficient downstream analysis.

Variant annotation

The SnpEff (v5.1) variant annotation tool [22] was employed to build a database for the reference genome GCF_000001405.40 (GRCh38.p14 database). Subsequently, SnpEff annotated variants via the GRCh38.p14 reference genome, predicting their functional effects on genes, transcripts, and protein sequences. This annotation process provided valuable insights into the potential functional consequences of variants within the context of coding regions and protein structures.

Furthermore, missense mutations with a moderate impact on protein function, as identified by SnpEff, were filtered via the point-accepted mutation or percent accepted mutation (PAM250) score, which was calculated via the Biostrings R package [23]. The scores reflect the likelihood of one amino acid being replaced by another during evolution. Higher scores indicate more probable substitutions, whereas lower scores indicate less likely substitutions. Only missense mutations with a PAM250 score less than one were retained. These missense mutations were also annotated via data from the SIFT [24], PolyPhen-2 [25] and ClinVar [26] databases through the SNPnexus [27].

Statistical analysis 

Statistical analyses were conducted using the R statistical computing environment (version 4.2.1, R Foundation for Statistical Computing, Vienna, Austria). Associations between genetic variants and clinical phenotypes were assessed using Spearman’s rank correlation via the cor.test() function. Due to the exploratory design of the study, p-values were not adjusted for multiple comparisons and should be considered nominal and hypothesis-generating. For the heatmap visualization, a correlation threshold of |ρ| > 0.6 was applied to highlight strong associations. Differences between groups were evaluated using the Mann-Whitney U test (wilcox.test()) with continuity correction.

## Results

Patient demographics and clinical characteristics

The present study aimed to discover novel genetic variants associated with FH in patients from Telangana, India. The study population comprised 30 patients from 15 families, with a mean age of 39 years (SD = 16). The gender distribution was skewed toward males, with 57% of the participants being male and 43% being female (Table 1 and Appendix 1).

**Table 1:** Summary statistics of the demographics and clinical characteristics of patients The study population (n = 30) had a mean age of 39 years (SD = 16), comprising 17 (56.7%) males and 13 (43.3%) females. The mean total cholesterol was 280 mg/dL (SD = 130), and the mean low-density lipoprotein cholesterol (LDL-C) was 238 mg/dL (SD = 135). A corneal arcus was present in nine (30%) participants, tendon xanthomas in six (20%), and family history of coronary artery disease was reported in 28 (93.3%) individuals.

Characteristic	Value (n = 30)
Age (years)	Mean(SD): 39± 16
Sex	Percent: M (57); F (43)
Total cholesterol (mg/dL)	Mean(SD): 280 ± 130
LDL-C (mg/dL)	Mean(SD): 238± 135
Corneal Arcus	30%
Tendon Xanthomas	20%
Family history of CAD	93%

Lipid profile analysis revealed significantly elevated levels of cholesterol in the study patients. The mean total cholesterol (TC) level was 280 mg/dL (SD = 130), which is well above the desirable range for cardiovascular health. Similarly, the mean LDL-C level was 238 mg/dL (SD = 135), indicating a substantial burden of atherogenic lipoproteins.

Clinical examination of the participants revealed the presence of characteristic signs associated with FH. A corneal arcus, a gray or white opaque ring around the cornea due to cholesterol deposition, was observed in 30% of the participants. Additionally, 20% of the participants presented with tendon xanthomas, which are localized deposits of cholesterol in tendons and are most commonly observed in the Achilles tendon.

Notably, a significant proportion (93%) of the patients reported a family history of coronary artery disease (FHx CAD), underscoring the genetic predisposition and familial nature of FH. This finding highlights the importance of comprehensive pedigree analysis in the diagnosis and management of FH.

The participants were further classified on the basis of the DLCNC, a widely accepted tool for diagnosing FH. The DLCNC scores categorize individuals into three groups: possible FH (DLNC > 3), probable FH (DLNC > 5), and definite FH (DLNC > 3). This classification system takes into account various factors, including lipid levels, physical signs and family history, to determine the likelihood of FH.

**Figure 1:**
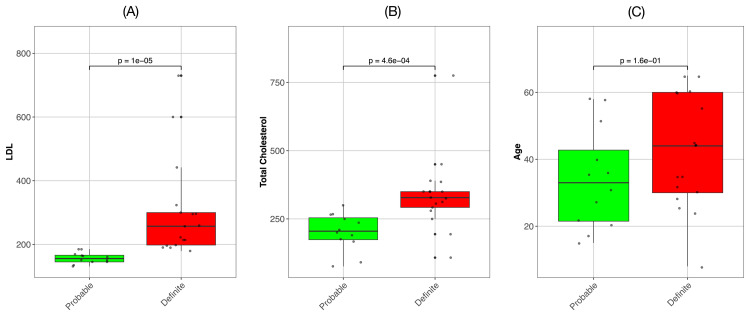
Box plots comparing the low-density lipoprotein (LDL), total cholesterol, and age distributions between individuals classified as having probable or definite familial hypercholesterolemia (FH) on the basis of their Dutch Lipid Clinic Network Criteria (DLCNC) scores. The statistical significance of differences in the means of two distributions was calculated via the Mann–Whitney U test. (A) LDL levels are significantly different between Probable FH and Definite FH. (B) Total cholesterol levels are significantly different between Probable and Definite FH. (C) The difference in age between probable and definite familial hypercholesterolemia patients was not significant.

As expected, LDL cholesterol levels were significantly higher in individuals with definite FH than in those with probable FH (p < 1e-05) (Figure 1). Similarly, total cholesterol levels were significantly different between the two groups, with definite FH cases exhibiting higher levels than probable FH cases (p < 4.6e-04). These findings suggest a strong correlation between FH severity, as determined by DLCNC scores, and elevated lipid levels.

Interestingly, we found no significant difference in age distribution between the Probable and Definite FH groups (p = 0.16). These findings suggest that age may not be a primary factor in differentiating between these two groups. Our findings imply that genetic factors play a more substantial role in determining the severity of FH, as evidenced by the distinction between Probable and Definite FH. For all the comparisons, we employed nonparametric statistical methods (Mann-Whitney U test) to account for the potential nonnormal distribution of the data.

The box plots with overlaid individual data points in our visualization (Figure 1) provide a comprehensive view of the data distribution, clearly illustrating the differences in lipid levels between the Probable and Definite FH groups, as well as the similarity in age distribution. These results support the validity of the DLCNC scoring system in differentiating between probable and definite FH on the basis of lipid profiles and highlight that age may not be a critical factor in this classification. This information may prove valuable for clinicians in understanding the relationships among FH severity, lipid levels, and patient age in the context of diagnosis and treatment planning.

Pedigree information for the study participants

Detailed pedigree information was collected for each of the 30 study participants, spanning 15 families. Each individual was identified by their unique family ID (FAM ID) and patient ID (PID). The sex of each participant was recorded (1 denoting male, 2 denoting female), along with their DLCNC category (Appendix 2).

The pedigree data included essential familial relationships, such as paternal ID (father), maternal ID (mother), and sibling IDs. This comprehensive information enabled us to construct detailed family trees and trace the inheritance patterns of FH within each family. By analyzing these pedigrees, we observed diverse patterns of FH transmission, including autosomal dominant inheritance, where the condition is passed down from one affected parent to their offspring, as well as sporadic cases where individuals developed FH without a clear family history.

Elucidating these familial patterns through detailed pedigree analysis is crucial for understanding the genetic basis of FH and for providing personalized risk assessments and genetic counseling to affected families. By identifying individuals at high risk of FH owing to their family history, early interventions such as lifestyle modifications and lipid-lowering therapies can be implemented to prevent or delay the onset of cardiovascular complications. Moreover, the identification of specific genetic mutations responsible for FH in certain families can facilitate targeted screening and treatment strategies.

High-impact variants identified in FH-associated genes

High-impact variants cause major disruptions to genes and proteins, often rendering them nonfunctional or severely altered. These variants can have a range of consequences, including the complete loss of gene function, the production of shortened proteins, or disruptions in the protein production process. For example, frameshift variants alter the reading frame of a gene, leading to a drastically different amino acid sequence. Nonsense variants introduce premature stop signals, resulting in the formation of truncated proteins. Splice site variants disrupt RNA processing, potentially causing errors in protein production.

Pedigree analysis revealed that the high-impact mutations identified in this study were distributed across multiple families, as detailed in Table 2. Targeted exome sequencing of the study population further revealed a range of high-impact genetic variants in genes previously associated with FH, as summarized in Table 3. Among the five high-impact mutations, four with different levels of clinical significance have already been reported in the dbSNP database [28].

**Table 2:** Pedigree information for familial hypercholesterolemia (FH) patients with high-impact mutations Each patient is identified by their family ID and patient ID, along with their sex (1: male, n = 11, 68.8%; 2: female, n = 5, 31.3%) and Dutch Lipid Clinic Network Criteria (2: probable FH, n = 8, 50%; 3: definite FH, n = 8, 50%). Relationships within families are indicated by paternal ID, maternal ID, and sibling IDs, elucidating the familial patterns of hypercholesterolemia within the study population.

Family ID	Patient ID	Pat. ID	Mat. ID	Sibling ID	Sex	DLCNC Cat
FAM01	F10	0	F9	F11	1	2
FAM02	F12	0	0	0	2	3
FAM03	F14	0	0	0	2	2
FAM05	F17	0	F18	0	1	2
FAM07	F1	F3	F2	0	1	3
FAM10	F23	0	0	0	1	2
FAM11	F24	0	0	0	1	2
FAM12	F25	0	0	0	1	2
FAM13	F26	0	F27	F28	1	2
FAM13	F27	0	0	0	2	3
FAM13	F28	0	F27	F26	2	3
FAM07	F2	F4	0	0	2	3
FAM07	F3	0	0	0	1	3
FAM07	F4	0	0	0	1	3
FAM14	F5	0	0	F6	1	3
FAM14	F6	0	0	F5	1	2

**Table 3:** High-impact variants identified in familial hypercholesterolemia (FH) associated genes. Stop-gain mutations were identified in LPL (6/30; 20%) and LDLR (4/30; 13.3%), splice donor site mutations in SLCO1B1 (1/30; 3.3%) and CETP (3/30; 10%), and a novel LDLR frameshift mutation in 2/30 (6.7%) was detected in siblings F5 and F6 from FAM14, with F5 carrying a homozygous variant (A/A, Definite FH) and F6 carrying a heterozygous variant (AC/A, Probable FH). The rs328 (LPL) variant is benign, rs769737896 (LDLR) is unreported in ClinVar, and rs571639279 (SLCO1B1) is of uncertain significance. The rs769737896 (LDLR) mutation in FAM07 showed heterozygous (C/T) in 2/4 patients (50%; F3, F4) and homozygous (T/T) in 2/4 patients (50%; F1, F2).

Family_ID	Patient ID	DLCNC Cat	LPL (rs328)	SLCO1B1 (rs571639279)	CETP (rs2142001776)	LDLR (rs769737896)	LDLR (Novel)
FAM01	F10	2	./.	G/A	./.	./.	./.
FAM02	F12	3	C/G	./.	./.	./.	./.
FAM03	F14	2	C/G	./.	./.	./.	./.
FAM05	F17	2	C/G	./.	./.	./.	./.
FAM07	F1	3	./.	./.	./.	T/T	./.
FAM10	F23	2	./.	./.	T/C	./.	./.
FAM11	F24	2	./.	./.	T/C	./.	./.
FAM12	F25	2	./.	./.	T/C	./.	./.
FAM13	F26	2	C/G	./.	./.	./.	./.
FAM13	F27	3	C/G	./.	./.	./.	./.
FAM13	F28	3	C/G	./.	./.	./.	./.
FAM07	F2	3	./.	./.	./.	T/T	./.
FAM07	F3	3	./.	./.	./.	C/T	./.
FAM07	F4	3	./.	./.	./.	C/T	./.
FAM14	F5	3	./.	./.	./.	./.	A/A
FAM14	F6	2	./.	./.	./.	./.	AC/A

We identified stop-gain mutations in two key genes involved in lipid metabolism, LPL (rs328, chr 8-19962213-C-G) and LDLR with heterozygous (rs769737896, chr 19-11110759-C-T) and homozygous stop gain mutations (chr 19-11110759-T-T) within the same family, FAM07. The variant rs328 is classified as benign, and another variant, rs769737896 (chr 19-11110759-C-T), resulting in p.Arg350Ter, is considered pathogenic in ClinVar [26]. The FinnGen database reports a significant association between the variant chr 8-19962213-C-G and various lipoprotein metabolism-related phenotypes, including statin medication usage, disorders of lipoprotein metabolism and other lipidemias, mixed hyperlipidemia, and pure hypercholesterolemia (https://r10.finngen.fi/). Stop-gain mutations introduce premature stop codons into gene sequences, resulting in the production of truncated proteins that are often nonfunctional or exhibit significantly reduced activity. In the context of the LPL and LDLR genes, LPL is crucial for hydrolyzing triglycerides within lipoproteins, and its dysfunction can lead to elevated triglyceride levels, potentially contributing to hypercholesterolemia. Moreover, LDLR is essential for the cellular uptake of LDL cholesterol, and its dysfunction results in elevated LDL-C levels in the bloodstream, a hallmark of familial hypercholesterolemia.

In addition to stop-gain mutations, we observed splice donor site mutations in two other genes, SLCO1B1 (rs571639279, chr 12-21141659-G-A) and cholesteryl ester transfer protein (CETP, rs2142001776, chr 16-56975153-T-C). The variant chr 12-21141659-G-A is classified as a variant of uncertain significance, and another variant, chr 16-56975153-T-C, is classified as likely pathogenic and has been associated with hyperalphalipoproteinemia 1 in the ClinVar database. Splice donor site mutations disrupt the normal splicing process of pre-mRNAs, often leading to the production of aberrant mRNA transcripts and potentially altered protein function. The SLCO1B1 gene encodes a protein called organic anion transporting polypeptide 1B1 (OATP1B1). OATP1B1 is a transmembrane receptor found in liver cells that transports compounds such as bilirubins and drugs from the blood into the liver for removal from the body. CETP is involved in the transfer of cholesterol esters between lipoproteins, and mutations in this gene can affect HDL cholesterol levels and overall cholesterol efflux capacity.

A key finding in our analysis was a novel frameshift mutation in the LDLR gene in two siblings (F5 and F6) from family FAM14. This mutation, characterized by a nucleotide deletion in chromosome 10 at position 11129663, was present in a heterozygous state (chr 10-11129663-AC/A) in F6 and a homozygous state (A/A) in F5. The presence of the homozygous mutation in F5 resulted in a "Definite FH" classification according to the DLCNC, underscoring the significant impact of this mutation on cholesterol metabolism and disease severity.

The identification of this novel frameshift mutation expands the known mutational landscape of LDLR in FH and contributes to our understanding of the genetic heterogeneity of this disorder. Further investigation into the functional consequences of this mutation will be crucial to determine its precise impact on LDLR function and cholesterol metabolism.

Moderate impact variants identified in FH-associated genes

Moderate impact variants are less likely to completely disrupt a gene or protein, but they can still lead to changes in function or effectiveness. For example, missense variants alter the amino acid sequence of a protein, whereas in-frame indels add or remove amino acids without shifting the reading frame. The specific impact of these changes can vary depending on the amino acid affected and its location within the protein.

**Figure 2:**
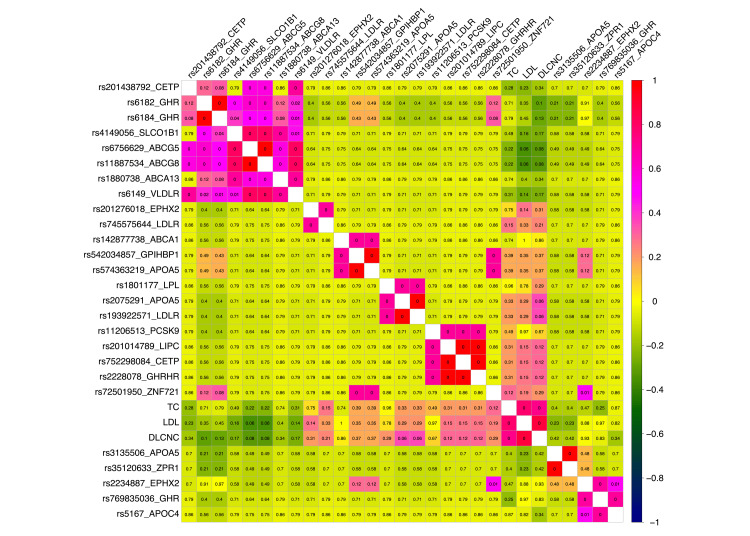
Correlation matrix heatmap showing Spearman correlations between genotype and phenotype variables. The correlation coefficients are represented by colors ranging from dark blue (strong negative correlation) through yellow (no correlation) to red (strong positive correlation). The variables are clustered hierarchically, with similar correlation patterns grouped together. Rectangles highlight major clusters of correlated variables. The P values of the correlation coefficients are displayed within each cell. The phenotype variables included low-density lipoprotein (LDL), total cholesterol, Dutch Lipid Clinic Network Criteria (DLCNC) score and age. Only correlations with an absolute value greater than 0.6 are included in this plot to focus on stronger associations. The single nucleotide polymorphisms (SNPs) rs2075291 (11-116790675-C-A, APOA5) and rs193922571 (10-11105268-G-A, LDLR) are strongly correlated with the DLCNC score, whereas the variants rs6756629 (2-43837950-G-A, ABCG5) and rs11887534 (2-43839107-G-C, ABCG8) are strongly negatively correlated with LDL and DLCNC.

Since it is challenging to assess the functional impact of moderate-effect variants directly on proteins, unlike high-impact variants, we conducted a correlation analysis between moderate-effect variants and various clinical phenotypes, including age, LDL, DLCNC score and total cholesterol levels across the 30 individuals. Figure 2 presents a comprehensive analysis of the relationships between genetic markers and clinical phenotypes.

We used Spearman rank correlation analysis to examine associations between genotype (SNP) data and key phenotypic variables. The p value significance of the correlations is provided in each cell of the heatmap. The SNPs rs2075291 (chr 11-116790675-C-A, APOA5) and rs193922571 (chr 10-11105268-G-A, LDLR) showed strong correlations with the DLCNC score, suggesting that these variants may increase susceptibility to conditions related to elevated cholesterol, such as familial hypercholesterolemia. Conversely, the variants rs6756629 (chr 2-43837950-G-A, ABCG5) and rs11887534 (chr 2-43839107-G-C, ABCG8) exhibited strong negative correlations with LDL and DLCNC, indicating that these variants could be protective, potentially contributing to lower cholesterol levels and reduced FH risk.

Further literature review revealed that the minor allele (A) of rs6756629 (chr 2-43837950-G-A, ABCG5) is associated with decreased LDL levels, increased HDL cholesterol levels, and decreased triglyceride levels [29]. In contrast, the G allele is associated with a lower risk of gallstone disease (cholelithiasis) [30]. Additionally, the C/C and GC variants of rs11887534 (chr 2-43839107-G-C, ABCG8) have been linked to an increased risk of gallstone disease in Egyptian patients, particularly among women who use hormones for various gynecological purposes.

To assess the impact of these mutations on protein function, we employed the PAM250 matrix, SIFT, PolyPhen-2 software, and data from the ClinVar database, as described in the methods section. The variant rs2075291 (chr 11-116790675-C-A, APOA5) was classified as benign or deleterious with low confidence and considered a risk factor, whereas rs193922571 (chr 10-11105268-G-A in LDLR) was likely pathogenic, probably damaging, and deleterious. In contrast, rs6756629 was considered benign, with low-confidence deleterious annotations, and likely benign, whereas rs11887534 was classified as possibly damaging or deleterious with low confidence, although it was also considered benign or likely benign and a risk factor.

The moderate-impact mutation at position 11105268 on chromosome 19 (rs193922571) in the LDLR gene (c.362G>A, p.Cys121Tyr) was predicted to be damaging by SIFT software and probably damaging by PolyPhen-2. It was also classified as likely pathogenic in the ClinVar database. Patients F7 and F8 from family FAM15 carried this mutation and had a DLCNC score of 3, suggesting a likely genetic cause of FH in these patients. Both patients were heterozygous (G/A), indicating that they may have had heterozygous autosomal dominant familial hypercholesterolemia.

## Discussion

This study provides valuable insights into the genetic landscape of familial hypercholesterolemia through targeted exome sequencing and pedigree analysis of 30 patients from 15 families. We identified a novel frameshift mutation in two (6.7%) siblings, one of whom (50%) exhibited a homozygous variant (chr 10-11129663-A/A) and a "Definite FH" classification according to the DLCNC, underscoring its severe impact on cholesterol metabolism and highlighting the importance of identifying novel pathogenic variants.

In addition to these novel findings, we also observed previously reported high-impact mutations, including stop-gain mutations in LPL (rs328, chr 8-19962213-C-G) and LDLR (rs769737896, chr 19-11110759-C-T and rs769737896, chr 19-11110759-T-T) and splice donor site mutations in SLCO1B1 (rs571639279, chr 12-21141659-G-A) and CETP (rs2142001776, chr 16-56975153-T-C), further emphasizing the genetic heterogeneity of FH. While some of these variants are classified as benign or of uncertain significance, their co-occurrence with FH phenotypes necessitates further investigation into their potential roles in disease pathogenesis. Additionally, the moderate-impact variants identified in this study, such as the missense mutation c.362G>A (p.Cys121Tyr, rs193922571, chr 10-11105268-G-A) in LDLR, were predicted to be damaging by in silico tools such as SIFT and PolyPhen-2 and were classified as likely pathogenic in ClinVar. Overall, these findings highlight the potential contribution of such variants to the FH phenotype, either independently or in combination with other genetic or environmental factors.

Our comprehensive pedigree analysis enabled us to trace familial inheritance patterns and identify individuals at high risk of developing FH. This information is crucial for implementing early interventions such as lifestyle modifications and lipid-lowering therapies to prevent or delay cardiovascular complications. Moreover, the identification of specific pathogenic mutations within families can guide targeted screening and treatment strategies, such as more aggressive interventions for individuals with novel LDLR frameshift mutations.

This study provides valuable insights into the genetic variants associated with FH in patients from Telangana, India; however, several limitations must be considered. The small sample size of 30 individuals from 15 families limits the generalizability of the findings. The study's focus on a specific regional population restricts its applicability to broader ethnic groups, and potential selection bias may have influenced the severity of FH observed. Addressing these limitations in future research through larger, multi-ethnic cohorts, functional validation studies, and longitudinal designs will improve our understanding of FH genetics and its clinical implications.

This study has several important limitations that must be acknowledged. The small sample size of 30 individuals from 15 families, while appropriate for an exploratory family-based study of a rare Mendelian disorder, significantly limits the statistical power for genotype-phenotype correlation analyses and prevents robust conclusions about variant frequencies or prevalence estimates in the broader population. The regional specificity of our cohort, restricted to Telangana State, further limits the generalizability of our findings to other Indian states or ethnic groups, which may harbor different mutational spectra due to founder effects, population stratification, and distinct genetic architecture. This geographic restriction means our findings may not be representative of the diverse Indian population or applicable to other South Asian populations. Consequently, the variant spectrum and frequencies identified in this study should be interpreted as preliminary observations specific to this regional cohort rather than definitive conclusions about FH genetics in India. Validation in larger, multi-ethnic cohorts from different geographic regions is essential to confirm our findings and establish their broader applicability.

## Conclusions

To conclude, this study successfully achieved its primary objective of characterizing the genetic variants associated with familial hypercholesterolemia in patients from Telangana State, India, through targeted exome sequencing and comprehensive pedigree analysis. The identification of both novel and known high-impact mutations, coupled with detailed pedigree analysis, provides preliminary insights into the genetic landscape of FH in this regional population. However, the generalizability of these findings is constrained by the small sample size and the lack of functional validation for the identified novel variants. While these results suggest potential implications for improved diagnosis, risk stratification, and personalized management of FH, validation in larger, multi-ethnic cohorts is essential before clinical implementation. The data generated from this exploratory study can contribute to future meta-analyses for identifying FH risk variants and developing polygenic risk scores for the Indian population.

## Appendices

Appendix 1 

**Table 4:** Patient demographics and clinical characteristics Patient demographics and clinical characteristics for 30 study participants, including age, sex (1: male, n=16, 53.3%; 2: female, n=14, 46.7%), total cholesterol (TC, mg/dL), low-density lipoprotein cholesterol (LDL, mg/dL), presence or absence of corneal arcus (CA; present: n=9, 30%; absent: n=21, 70%) and tendon xanthomas (TX; present: n=6, 20%; absent: n=24, 80%), family history of coronary artery disease (FHx CAD; yes: n=28, 93.3%; no: n=2, 6.7%), and Dutch Lipid Clinic Network Criteria (DLCNC) score and category (1: possible Familial Hyper Cholesterolomia (FH), n=1, 3.3%; 2: probable FH, n=16, 53.3%; 3: definite FH, n=13, 43.3%).

Patient ID	Age	Sex	TC	LDL	CA	TX	FHx CAD	DLCNC	DLCNC Cat
F10	22	1	176	131	0	0	1	6	2
F11	20	2	167	135	0	0	1	6	2
F9	55	2	306	214	0	0	1	8	3
F12	65	2	450	323	1	0	1	12	3
F13	32	2	250	190	0	0	1	8	3
F14	44	2	312	214	0	0	1	8	2
F15	31	1	236	169	0	0	1	6	2
F16	51	2	267	145	0	0	1	6	2
F17	35	1	328	222	0	0	1	8	2
F18	60	2	350	190	0	0	1	8	2
F19	28	1	292	179	0	1	0	11	3
F20	58	1	203	154	0	0	1	4	1
F1	8	1	775	730	1	1	1	20	3
F2	24	2	350	442	1	1	1	20	3
F3	30	1	325	259	1	0	1	11	3
F4	60	1	390	295	1	1	1	15	3
F21	35	1	350	257	0	0	1	8	2
F22	58	2	300	185	0	0	1	6	2
F23	35	1	250	160	0	0	1	6	2
F24	40	1	210	151	0	0	1	6	2
F25	58	1	190	150	0	0	1	6	2
F26	36	1	266	185	0	0	1	6	2
F27	65	2	108	196	1	0	1	10	3
F28	45	2	194	296	0	0	1	8	3
F29	17	1	91	164	0	0	1	6	2
F30	15	2	76	145	0	0	1	6	2
F5	25	1	350	600	1	1	1	20	3
F6	27	1	200	165	0	0	1	6	2
F7	44	1	386	300	1	1	0	20	3
F8	60	2	280	198	1	0	1	12	3

Appendix 2 

**Table 5:** Pedigree information for the 30 study participants across 15 families. Each patient is identified by their family ID and patient ID, along with their sex (1: male, n=16, 53.3%; 2: female, n=14, 46.7%) and Dutch Lipid Clinic Network Criteria (DLCNC) category (1: possible Familial Hyper Cholesterolomia (FH), n=1, 3.3%; 2: probable FH, n=16, 53.3%; 3: definite FH, n=13, 43.3%). Relationships within families are indicated by paternal ID, maternal ID, and sibling IDs (0 = not available/not applicable), elucidating the familial patterns of hypercholesterolemia within the study cohort.

Family ID	Patient ID	Paternal ID	Maternal ID	Sibling ID	Sex	DLCNC Cat
FAM01	F10	0	F9	F11	1	2
FAM01	F11	0	F9	F10	2	2
FAM01	F9	0	0	0	2	3
FAM02	F12	0	0	0	2	3
FAM02	F13	0	F12	0	2	3
FAM03	F14	0	0	0	2	2
FAM04	F15	0	F16	0	1	2
FAM04	F16	0	0	0	2	2
FAM05	F17	0	F18	0	1	2
FAM05	F18	0	0	0	2	2
FAM06	F19	F20	0	0	1	3
FAM06	F20	0	0	0	1	1
FAM07	F1	F3	F2	0	1	3
FAM07	F2	F4	0	0	2	3
FAM07	F3	0	0	0	1	3
FAM07	F4	0	0	0	1	3
FAM08	F21	0	0	0	1	2
FAM09	F22	0	0	0	2	2
FAM10	F23	0	0	0	1	2
FAM11	F24	0	0	0	1	2
FAM12	F25	0	0	0	1	2
FAM13	F26	0	F27	F28	1	2
FAM13	F27	0	0	0	2	3
FAM13	F28	0	F27	F26	2	3
FAM13	F29	0	0	F30	1	2
FAM13	F30	0	0	F29	2	2
FAM14	F5	0	0	F6	1	3
FAM14	F6	0	0	F5	1	2
FAM15	F7	0	F8	0	1	3
FAM15	F8	0	0	0	2	3
